# 26/11 Mumbai terrorist attack revisited: Lessons learnt and novel disaster model for future

**DOI:** 10.4102/jamba.v12i1.915

**Published:** 2020-08-24

**Authors:** Dhiraj V. Sonawane, Bipul K. Garg, Ajay Chandanwale, Ambarish A. Mathesul, Omkar R. Shinde, Shravan Singh

**Affiliations:** 1 Department of Orthopaedics, Grant Medical College and Sir J.J. Group of Hospitals, Mumbai, India; 2 Department of Orthopaedics, Sassoon General Hospital and Byramjee Jeejeebhoy Medical College, Pune, India

## Introduction

Terrorism is the unlawful exercise of random and ruthless violence against property or individuals, usually innocent civilians, in order to intimidate governments or societies for political or ideological purposes. Mumbai, the financial capital of India and its busiest metropolitan city, has been a prime target for terrorist attacks. In the last two decades, the number of terrorist attacks in Mumbai have caused over 700 fatalities (Table 1). On 26 November 2008, ten transnational terrorists attacked Mumbai, which included the busiest railway station in peak hour, five-star hotels, a café shop and hospitals. The multiple attacks and control measures lasted for three days, leading to the deaths of over 149 people which included civilians, foreign nationals, security personnel and hospital staff. The attack was a meticulously planned and executed act of terrorism where explosive devices and gunfire were used to cause the maximum number of casualties and lasted for 60 h. This attack was therefore different from previous attacks which were serial blasts in Mumbai in 2006 (Hirshberg, Holcomb & Mattox 2001), and in London in 2005 (Aylwin et al. 2006; Bhandarwar et al. 2012). In the 2011 attack, 66.8% of injured people required surgical interventions, compared to less than 35% in both the Mumbai and London blasts (Deshpande, Mehta & Kshirsagar 2007). The Sir J.J. Group of Hospitals (SJJGH) in Mumbai received the maximum number of casualties (271 patients) in a short duration of time. The modes of transport of patients were mainly taxis, handcarts, fire brigade vans, ambulances and private vehicles, assisted by local people. The in-hospital disaster plan was activated immediately, as large numbers of patients were expected.

**TABLE 1:** Number of casualties and mortalities in terror attacks in Mumbai over the last two decades.

Sl no.	Date	Place	Killed	Injured
1	12 March 1993	13 blasts across the city	257	713
2	28 August 1997	Near Jama Masjid	0	3
3	24 January 1998	Malad	0	1
4	27 February 1998	Virar	9	0
5	2 December 2002	Ghatkopar	3	31
6	6 December 2002	Mumbai Central Railway Station	0	25
7	27 January 2003	Vile Parle	1	25
8	13 March 2003	Mulund Railway Station	11	80
9	14 April 2003	Bandra	1	0
10	29 July 2003	Ghatkopar	3	34
11	25 August 2003	Gateway of India and Zaveri Bazaar	52	160
12	11 July 2006	7 blasts at 7 locations in local trains across the city	181	890
13	26 November 2008	Multiple terrorist attacks across the city	166	300
14	13 July 2011	Serial blasts in Mumbai	26	131

**Total**			**710**	**2393**

*Source*: South Asia Terrorism Portal, 2001, *Terrorist attacks in Mumbai since 1993,* Institute for Conflict Management, viewed 05 May 2019, from http://www.satp.org/satporgtp/countries/india/database/mumbai_blast.htm#

Sl, serial.

This study describes the pattern of injuries during the attack and the distribution of patients in various hospitals. Two fundamental aims of disaster management are the rapid evacuation of all casualties from a hazardous incident scene, and the reduced mortality of critically injured patients. The purpose of this study was to identify the medical response time to the terrorist attack, identify lacunae in disaster mitigation, suggest organised approaches to save lives and limit disability, and give detailed measures towards better preparedness in dealing with future terror strikes for Indian metropolitan cities.

## Materials and methods

### Study design

This is a retrospective study.

### Materials

Data of all patients presenting at various hospitals, including SJJGH, Bombay Hospital, Cooper Hospital, Gokuldas Tejpal Hospital, KEM Hospital, Nair Hospital and St. George Hospital, who had injuries sustained during the 26/11 terrorist attack, were included in the study.

### Methods

A retrospective analysis of patients’ data from all of the hospitals mentioned above was carried out. This study was conducted at SJJGH, which serves as the definitive receiving facility. Data were collected by a thorough review of the inpatient and outpatient records. The patients’ data were studied under the following sub-headings: age, sex, diagnosis, prior care received, number and types of procedure, and number of patients triaged at the various hospitals. The data were analysed to learn about medical response interactions and the main outcomes.

### Ethical consideration

The study was initiated after approval was received from the J.J. Group of Hospitals and Grant Medical College Research and Ethical Committee.

## Results

The majority of patients (271) were managed at the SJJGH, which was 3.5 km/8 minute distance from the disaster site, although other hospitals, such as the St. George Hospital, which was 900 m/2 minutes distance; the GT Hospital, which was 950 m/2 min distance; and the Bombay Hospital, which was 2 km/5 min distance from the incident site, managed 38, 30 and 79 patients respectively. The detail of the number of patients managed and the distance of the hospital from the disaster site are indicated in Table 2.

**TABLE 2:** Detail of the number of patients managed and the distance of the hospital from the disaster site.

Hospital	Number of patients	Diagnosis	Distance/time from site
Sir J.J. Group of Hospitals	271	108 dead/163 injured	3.5 km/8 min
Bombay Hospital	79	4 dead	2 km/5 min
Cooper hospital	5	3 dead	2.9 km
GT Hospital	30	11 dead	950 m/2 min
KEM Hospital	4	-	7 km/12 min
Nair Hospital	6	3 dead	5 km/9 min
St George Hospital	38	14 dead	900 m/2min

GT Hospital, Gokuldas Tejpal Hospital; KEM Hospital, King Edward Memorial Hospital.

The total number of interventions performed at the Sir J.J. Group of Hospitals was 194. These interventions involved: local exploration in 47 patients, primary closure of contused lacerated wounds in 30 patients, laparotomy in 22 patients, internal fixation in 19 patients, insertion of an intercostal drain in 15 patients, external fixation in 13 patients, foreign body removal in 13 patients, skin grafting in 13 patients, secondary wound closure in 6 patients, amputation in 4 patients, flap in 4 patients, craniotomy in 2 patients, tendon repair in 2 patients, vascular repair in 2 patients, thoracotomy in 1 patient and tracheostomy in 1 patient.

## Discussion

This terror attack has shown that Mumbai lacked pre-hospital care and on-scene triage. Previous attacks have shown a lack of coordination and poor transport facilities in managing disasters. During disasters, local bystanders have played a major role in the transport of casualties. These attacks have challenged the ability of disaster preparedness regarding medical response, security and infrastructure. The attacks have been frequent and have targeted crowded areas of the city with the aim of causing maximum damage. The recent 2011 Mumbai terror attacks were the deadliest terror strike. On retrospective evaluation of the data, some obvious loopholes were evident in handling this terrorist attack. Mumbai lacks the means to manage this disaster in providing emergency medical care especially at a pre-hospital level. This was compounded by a lack of coordination and poor transportation infrastructure. The majority of injured patients were brought to the hospital by local people and passers-by, using handcarts, taxis and private vehicles. Hundreds of patients were received at SJJGH in a short time frame, which overloaded the hospital. Although other trauma centres were kept on standby, they received few patients. Consequently, only one hospital was overloaded by the victims of this terror attack (Table 2).

Roy et al., in their retrospective descriptive study on the Mumbai terror attacks, concluded that there was a need to build a central medical control and strengthen public hospital capacity, and also that a formal emergency medical services (EMS) system and triage is absent in the city of Mumbai (2011). They found that the distribution of victims was determined by the proximity of the hospital to the blast site and the type of pre-hospital transport, and these occurred without field triage. Pinkert et al. studied the January 2006 Tel Aviv bomb blast in Israel and described it according to the Disastrous incidents systematic analysis through components, interactions and results (DISAST-CIR) methodology (2008). They concluded that in the event of mass casualty, the incident necessitated primary triage, evacuation priority decision- making, and the rapid distribution of casualties between all the adjacent hospitals, which would enable efficient and effective containment of the event.

The average/mean incident-to-arrival time in our study was 21 hours (2 h to 44 h) for major injuries and 14 h (30 min to 39 h) for minor injuries, which was more than the time taken in disaster and terrorism-prone countries like Israel. Also, as per our study and previous reports of the Mumbai terror attacks, there was an unequitable distribution of patients amongst the major public hospitals. Had there been a comprehensive disaster module, involving triage, field teams and transport teams, the number of casualties could have been reduced.

### Our proposal

**FIGURE 1:**
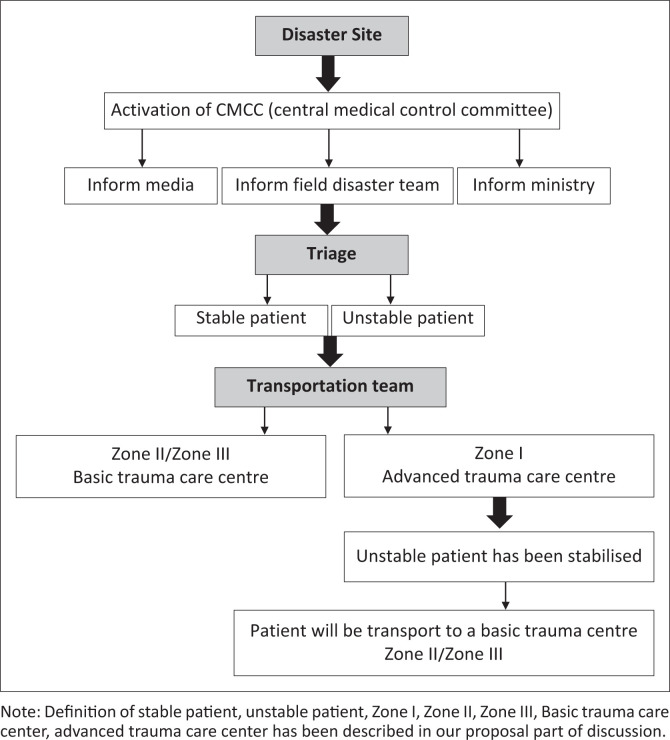
Disaster mitigation protocol.

We feel the need for the development of a comprehensive disaster module for the city of Mumbai and for Indian cities overall. The module would function as described in Figure 1 and consist of the formation of a central medical control committee (CMCC): This body would comprise of experts of tertiary medical care centres or experienced personnel who are capable of handling trauma and mass casualties, who would be responsible for handling the response to a terror attack or disaster.

Functions of the CMCC body would involve:

- An efficient and timely response to a disaster.
- Coordination with the ministry.
- The upgrading of available infrastructure.
- The training of staff and carrying out mock drills.
- Informing the media.

The present infrastructure (existing trauma care centres) and city need to be graded into levels:

- Advanced trauma care centre: Public hospitals with medical colleges within the city limits.
- Basic trauma care centre: Other public trauma care centres within the city limits.

Zones: With disaster sites as the focal point, trauma care centres falling within a particular time/distance from the disaster would be divided into the following categories:

- Less than 5 km/30 min from the disaster site/nearest the advanced trauma care centre.
- Within 5–10 km/30 min – 2 h of the disaster site.
- Greater than 10 km/2 h from the disaster site (by road via available vehicle as per Google Maps).

The CMCC body, with the available infrastructure and workforce comprising hospital authorities would need to direct and coordinate the response with other major trauma centres in the city, which would act according to the pre-planned disaster management protocol. This should ensure the equitable distribution of the casualties and efficient management.

We would like to suggest a disaster mitigation protocol for such future disasters with emphasis on pre-hospital care (Figure 1). In pre-hospital management, the cooperation of local by-standers as well as police, public servants, taxi drivers, bus drivers and their vehicles is imperative. These motivated by-standers, with minimal formal training, would play an active role in a ‘play and run method’ of management of the victims. In our protocol, we divided patients into two broad categories, which are (1) urgent unstable patients with injury to vital organs, spontaneous pneumothorax, internal bleeding, a stab wound in the trunk, burns > 10%, hypovolaemic shock, compound fracture with significant haemorrhage, spine injury, et cetera and (2) stable patients with close fractures, contused lacerated wound, minor injuries, and patients remaining unstable after having been stabilised.

## The composition and role of the Central Medical Control Committee

### Teams

- Field disaster team

### Personnel

- Medics (trauma specialist, physicians, anaesthetist)
- Paramedics (nurses, trainee)
- Hospital staff (ward boys, drivers, etc.)
- Bystanders

### Equipment

- Mobile van (dressing material, splints, IV fluids, emergency kit, etc.)

### Transport team

- Mobile intensive care unit
- Physician, anaesthetist
- Paramedics

### Emergency medical services team at hospital

- Triage at entrance by trauma specialist
- Management according to advanced trauma life support protocol
- Team approach consisting of the surgeon, orthopaedician, anaesthesiologist and specialist
- Documentation and communication

## Conclusion

Proper planning, a disaster management protocol and preparedness for such disaster can use these resources to their optimum level at the time of a disaster, to enable the timely management of injured patients who can be given treatment based upon severity, haemodynamic instability and the proximity of effective health care for prompt early treatment, without an excessive load being placed on selected hospitals, to reduce critical mortality.
